# Protective Effect of Vitamin C and Zinc as an Antioxidant Against Chemotherapy-Induced Male Reproductive Toxicity

**DOI:** 10.25122/jml-2019-0107

**Published:** 2020

**Authors:** Toktam Hajjar, Foroogh Soleymani, Mehran Vatanchian

**Affiliations:** 1. Department of Biology, Faculty of Sciences, Hakim Sabzevari University, Sabzevar, Iran.; 2. Department of Anatomical Sciences, School of Medicine, North Khorasan University of Medical Sciences, Bojnurd, Iran.

**Keywords:** Antioxidant, male infertility, Vitamin C, Zinc, chemotherapy

## Abstract

Treatment with anticancer drugs such as cyclophosphamide can harm the male reproductive system. Vitamin C and zinc are micronutrients with antioxidant activity and are the essential components of semen. Therefore, this study aimed to evaluate whether cyclophosphamide-exposed mice can recover from fertility with vitamin C and zinc therapy.

In this experimental study, fifty male mice were divided into five groups. Groups 1-4 received cyclophosphamide (100 mg/kg, once a week for eight weeks). Also, group 2 received zinc (200 mg/kg), group 3 received vitamin C (300 mg/kg), group 4 received zinc and vitamin C (200 mg/kg and 300 mg/kg, respectively), five times per week for eight weeks, and group 5 received normal saline once a week and water five days a week for eight weeks. The data collected were statistically analyzed using SPSS 22.

Results showed a significant increase in mount latency and a significant decrease in the number of sperms in the cyclophosphamide group compared to the control group. However, mount latency has been significantly decreased in mice treated with cyclophosphamide plus zinc compared to the cyclophosphamide group. The study also showed that the sperm count in the group that received cyclophosphamide and zinc had been increased compared to the cyclophosphamide group; the other treatments have decreased mount latency and increased the sperm count compared to the group treated with cyclophosphamide but not significantly. The Tubule Differentiation Index showed an increase in the cyclophosphamide-Zinc-Vitamin C group in comparison with the cyclophosphamide group.

The current study showed that zinc could improve cyclophosphamide-induced toxicity of the reproductive system in male mice.

## Introduction

Many infertility cases can be explained by male-only factors [1]. Male-related factors that cause infertility can be an alteration in sperm concentration, sperm morphology, and motility [2], and these abnormalities have dietary, environmental, genetic, or medical origins [3]. Medical factors such as drugs, chemotherapy, and radiotherapy can increase the production of intracellular reactive oxygen species (ROS) [4]. ROSs have a significant effect on spermatogenesis and sperm quality. An imbalance between ROS levels and physiologic antioxidants can result in oxidative stress, with subsequent adverse impact on the reproductive system [5].

Most of the chemotherapeutic drugs cause various types of damage to healthy living cells. Cyclophosphamide (CP) exhibits potent anticancer effects, and CP therapy is a common problem in the treatment of a variety of diseases and leads to gonadal toxicity as a side effect of the drug [6]. Previous studies have shown that CP increases abnormal sperm rate, and manifest biochemical and histological alterations in testis [7]. CP toxicity in the testis and spermatozoa is caused by oxidative stress-induced biochemical and physiological damage [8].

The studies have shown that some nutritional therapies improve sperm counts and sperm motility. These include arginine, zinc, selenium, and vitamin C [9]. Vitamin C (Vit C) has been found to help in preventing cell damage by neutralizing free radicals [10].

Antioxidants like vitamin C (ascorbic acid) has been proven to ameliorate the oxidative stress and sperm toxicity induced by endosulfan in rats [11]. The protective effects of vitamin C on genotoxicity and cytotoxicity have also been confirmed in mice [12]. Vitamin C comprises 65% of the antioxidant capacity of semen and is the most critical seminal antioxidant [13]. However, a study showed that vitamin C has controversial effects on sperm parameters and pregnancy rate in subfertile males with idiopathic oligoasthenoteratozoospermia [14]. Vitamin C is a powerful antioxidant, and it has been found to enhance sperm quality and prevent sperm agglutination, thus making them more motile with more forward progression [15, 16].

Because the effect of vitamin C in spermatogenesis is still controversial, this study tends to evaluate the role of supplemental vitamin C as an antioxidant nutrient after chemotherapy with CP for its probable improvement of sperm quality.

Zinc is a critical nutrient for proper male hormone metabolism and sperm production [9]. On the other hand, studies have shown that CP exposure significantly reduced the levels of zinc (Zn) in the serum and testes [17]. Since Zn is an essential element required for the maintenance of germ cells, progression of spermatogenesis, and regulation of sperm motility, it was found that supplementation with this element protects against CP-induced testicular damage [18]. Zinc deficiency can lead to impotence, and zinc therapy may improve sexual performance [19]. Previous studies have found that zinc levels are lower in infertile men with diminished sperm count, and supplemental zinc is useful in treating male infertility [20]. Zinc has an antioxidative activity and plays a vital role in the scavenging of ROS [21]. Thus, Zn deficiency is associated with increased oxidative stress and subsequent oxidative damage, such as low sperm quality [22]. The rats fed a zinc-deficient diet have shown a significant increase in the malondialdehyde (MDA) level, and a decrease in glutathione (GSH) content and superoxide dismutase (SOD) activity, and zinc supplementation reversed these effects [23].

The current study attempts to investigate the antioxidative effect of Zn and vitamin C on sperm quantity and determine whether these micronutrients could cause deleterious changes in sperm count and promote behavioral alterations in male mice models with cyclophosphamide-induced reproductive damage.

## Material and Methods

### Animals

In this experimental study, fifty healthy adult male mice (8 weeks old) were used. The animals were obtained from the Razi Vaccine and Serum Research Institute and were housed under standard laboratory conditions (temperature 24 ± 3°C, humidity 40–60%, 12-hour light-dark cycle). A commercial feed pellet and fresh drinking water were offered ad libitum.

The animals were randomly divided into five experimental groups of ten rats each. CP was administered to the animals at a dose of 100 mg/kg once a week for eight weeks, intraperitoneally. The groups were arranged as follows:

• Group 1 – CP: received CP and water as a placebo by gavage for five days a week for eight weeks.
• Group 2 – CP and Zn: treated with CP and zinc at a dose of 200 mg/kg by gavages for five days a week for eight weeks.
• Group 3 – CP and Vit C: treated with CP plus vitamin C at a dose of 300 mg/kg by gavage for five days a week for eight weeks.
• Group 4 – CP, Zn and Vit C: received CP plus a mixture of zinc (200 mg/ kg) and vitamin C (300 mg/kg) by gavage for five days a week for eight weeks.
• Group 5 – Control: received standard saline solution once a week and water five days a week for eight weeks intraperitoneally and orally, respectively.

### Sexual behavior assessment

After the treatment period, control and treated male rats with no previous sexual experience were placed in a Plexiglas cage for analysis of masculine sexual behavior. The test was done under dim red lights, two hours after the onset of the dark phase of the light-dark cycle. Masculine sexual behavior was assessed by placing the male 5 minutes before a female was presented. After the presentation of the female rat, the tests lasted 30 minutes, and the following parameters were recorded: the mount number, number of intromissions, latency to the first mount, and latency to the first intromission.

### Hormonal assay

After the sexual behavior assessment, all mice were anesthetized using ketamine/xylazine. Blood was collected, and plasma was separated by centrifugation and stored at -20°C for testosterone determination by radioimmunoassay.

### Sperm count

The cauda epididymis was removed and was punctured with a needle; a mass of sperm was squeezed out into a Petri dish containing 1 ml of phosphate-buffered saline (PBS, pH 7.2). The sperm suspension was poured into a tube, and the tube was immediately placed into the incubator at 37°C. After 30 minutes, the sperm suspension of 0.5 ml was diluted with 9.5 ml PBS. The dilution was mixed thoroughly and charged into Neubauer’s chamber and covered with a coverslip and viewed under a light microscope. The sperm count was conducted in eight randomly picked boxes from the counting chamber. The total count was then multiplied by the correction factor.

### Histological Preparation

Left testes were rapidly removed and fixed in 10% buffered formalin solution for 48 hours, dehydrated in a graded series of ethanol and embedded in paraffin. Thin sections (5-6 μm) were stained with hematoxylin and eosin and examined using a light microscope (Olympus, CX41). A total of 250 seminiferous tubules were analyzed microscopically, and the germinal epithelium height and tubule differentiation index (TDI) were obtained, the percentage of seminiferous tubules containing at least three generations of spermatogenic cells [24].

### Statistical analysis

The statistical interpretation for parametric data (sperm counts data) was made using one-way ANOVA followed by Tukey’s or Dennett’s Post Hoc test to find out whether the variances of groups were equal or not, using SPSS 22. Data with non-parametric distribution (behavioral and hormone data) were analyzed using the Kruskal-Wallis test, followed by Dunn’s test if multiple comparisons were needed, using the R statistical software. The significance level of tests was considered as 5%. All the data are expressed using the mean ± the standard error (SE) of the mean.

## Results

### Sexual behavior test

**Figure 1:**
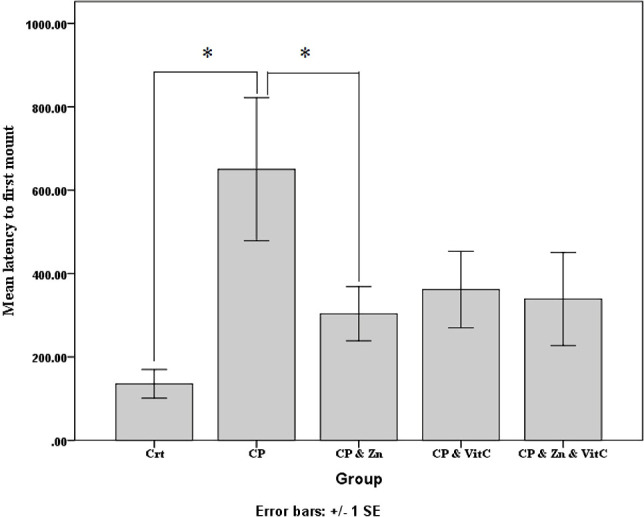
Mount latency (S) in all groups. The latency to first mount increased significantly in the CP group, but the CP and Zn group showed a significant decrease this time as compared with the CP group. Data are shown as Mean ± SE. *p <0.05, significant differences between groups.

As shown in Figure 1, compared to control animals, CP-treated mice (CP group) exhibited a significant increase in first mount latency (p<0.05). Moreover, Zn decreased mount latency significantly (p<0.05) in mice treated with CP and Zn compared to the mice that received only CP. The mice supplemented with vitamin C also showed a decreased mount latency compared to those that received only CP, but not significantly (p>0.05). Figure 1 indicates that the CP, Zn and Vit C group did not show a significant decrease in mount latency in comparison to the CP group either (p>0.05).

The results also showed there were no significant differences between groups regarding other items such as the mount number, number of intromissions, or latency to the first intromission (Table 1).

**Table 1:** Mount number, number of intromissions and intromission latency (S) in all groups (Mean ± SE).

**Groups**	**Mount number**	**Number of intromissions**	**Intromission latency (S)**
**Control**	7.00 ± 1.51	7.37 ± 2.76	580.62 ± 155.44
**CP**	3.62 ± 0.80	3.12 ± 1.20	909.75 ± 86.87
**CP and Zn**	2.87 ± 0.55	1.37 ± 1.01	31035.62 ± 108.60
**CP and Vit C**	4.00 ± 0.71	0.87 ± 0.39	977.12 ± 121.82
**CP, Zn and Vit C**	5.00 ± 4.40	2.20 ± 1.35	1089.60 ± 83.13

Note: CP - Cyclophosphmide, Zn - Zinc, Vit C - Vitamin C

### Hormone analysis

The effects of CP, zinc and vitamin C on the testosterone level are presented in Table 2. The results showed that chemotherapy decreased the level of this hormone in the CP group compared to the control group, but the reduction is not significant (p>0.05). As shown in Table 2, the plasma level of the hormone in the CP and Zn, CP and Vit C and CP, Zn and Vit C groups have been increased compared to the CP group, but not significantly (p>0.05).

**Table 2:** The level of testosterone (ng/dl) in all groups (Mean±SE).

**Groups**	**Hormone level**
**Control**	2.40 ± 0.61
**CP**	0.8 7± 0.54
**CP and Zn**	1.79 ± 0.77
**CP and Vit C**	2.06 ± 1.01
**CP, Zn and Vit C**	1.22 ± 1.12

Note: CP - Cyclophosphmide, Zn - Zinc, Vit C - Vitamin C

### Sperm count

**Figure 2:**
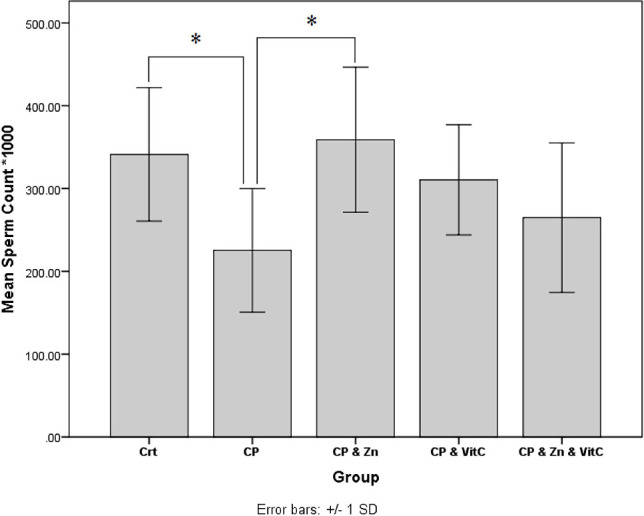
Effect of CP, Zn and vitamin C on sperm count. The number of sperms decreased significantly in the CP group, but the CP and Zn group showed a significant increase in sperm count as compared with the CP group. Data are shown as Mean ± SE. *p <0.05, significant differences between groups.

The effects of cyclophosphamide and different treatment on total sperm count are presented in Figure 2. The results showed that chemotherapy significantly decreased the sperm count in the CP group compared to the control group (p<0.05). As shown in Figure 2, the number of sperms in the CP and Zn group has been significantly increased compared to the CP group (p<0.05). The results also showed that the number of sperms in the CP and Vit C, as well as CP, Zn and Vit C groups showed an increase compared to the CP group but not significantly (p>0.05).

### Histological Findings

As shown in Table 3, germinal epithelium height increases very little (from 65.75 ± 2.99 in the control group to 70.81 ± 2.01 in the CP group). Changes of the germinal epithelium height between the groups were not significant. A comparison of the tubule differentiation index (TDI) between groups showed that TDI came back to the optimum level in the CP, Zn and Vit C group, but not significantly.

**Table 3:** Germinal epithelium height and TDI in all groups (Mean±SE).

**Groups**	**Germinal epithelium height (μm)**	**TDI (%)**
**Control**	65.75 ± 2.99	93.75
**CP**	70.81 ± 2.01	78.12
**CP and Zn**	68.12 ± 2.99	78.12
**CP and Vit C**	63 ± 2.58	81.25
**CP, Zn and Vit C**	69.05 ± 3.79	95

Note: CP - Cyclophosphmide, Zn - Zinc, Vit C - Vitamin C

## Discussion

One of the main aims of the present study was to answer whether chemotherapy as a stressor may alter sexual behavior. Compared to controls, CP-treated mice exhibited an increased latency for the first mount that might be suggestive of a lower sexual motivation [25]. Many neurotransmitters and neuropeptides play a role in the control of male sexual behavior. An increase in the activity of brain noradrenergic and dopaminergic correlates with the improvement of parameters of copulatory activity [26]. The effect of dopamine on male sexual behavior occurs by interacting with testosterone [27; 27]. Also, a proper androgenic status is also necessary for normal sexual performance [26]. The oxidative stress may be a mechanism that mediated the downregulation of testicular steroidogenesis and reduction of testosterone levels in CP-exposed mice. Thus, impairment of testicular steroidogenesis might associate with the generation of large amounts of ROS in testicular tissue [29], leading to sexual activity reduction.

Chemotherapy, as an exogenous factor (like pesticides and environmental pollutants), has negative effects on testicular function by inducing oxidative stress, while consumption of antioxidants has demonstrated to protect the testicular function in animal models [30].

In our study, the sperm count decreased after chemotherapy compared to the control animals; the administration of zinc was effective on sperm count and provided significant protection against CP-induced reductions in the number of sperms. However, no statistically significant differences in the sperm count in the vitamin C-treated mice or zinc and vitamin C-treated mice were observed, indicating that an antagonistic effect between vitamin C and zinc may exist.

We also observed that CP decreased the plasma testosterone level; this may be due to the abnormality of Leydig cells caused by oxidative stress [31]. Zinc administration could improve the antioxidative status and testosterone levels by increasing the concentration of zinc in seminal plasma and serum [32]. In the present study, zinc and vitamin C did not affect testosterone plasma levels. However, previous studies have shown that zinc–curcumin dose-dependently enhanced the level of testosterone in the testis of CP-treated mice. This suggests that the lower concentration of zinc (lower than 200 mg/kg) supplementation may alleviate the CP-induced reduction in the testosterone level more potently. The free radical scavenging action of zinc in the testis and the ability to protect Leydig cells are mechanisms that increase the testosterone level in animals treated with CP, and our results indicate that these mechanisms may be dose-dependent.

On the other hand, vitamin C supplementation had positive effects on sperm count and hormone level in male mice treated with CP; but it was not significantly effective. This means that Vitamin C could positively affect the quantitative characteristics of sperm analysis, and maybe higher doses of ascorbic acid used could result in a significant increase in these parameters. Previous studies that used the same dose of Vitamin C showed that it could increase motility and normal morphology after varicocelectomy surgery but not sperm count. These findings confirmed our results [33].

The evaluation of the effects of different dosages of vitamin C on the qualitative and quantitative characteristics of sperm needs more elaborate research; however, one study has shown that an increase in vitamin C dosage to 1000 mg per day significantly improved these parameters in smokers [34].

One study on mice showed that higher doses of vitamin C could prevent sperm toxicity induced by pesticides and improve the sperm count and morphology after pesticide exposure [35]. Another study proved that water-soluble vitamins improve semen quality and increase motile sperm counts in boars [36].

Animal studies have shown that vitamin C can reduce the oxidative stress induced by various factors and decrease the number of abnormal sperms [37, 38]. A study on humans has also illustrated that vitamin C and vitamin E administration in men with a high percentage of DNA-fragmented spermatozoa decreased the fragmentation of DNA significantly [39].

Crocin (10 and 20 mg/kg) was able to repair diminished germinal epithelium height induced by CP (p<0.05 and p<0.01, respectively) [40]. Zinc oxide nanoparticles (ZnONPs) (5 mg/kg, daily for eight weeks), increased germinal epithelium height from 207 μm in the CP group to 250.17 μm (p<0.001) [41].

Also, Spirulina platensis (3 mg/kg and 30 mg/kg) has been able to change TDI from 12% in the CP group to 66.33 and 72%, respectively, which shows a relatively good condition compared to the control group (95%) [42]. In this study, CP was injected only once a week compared with long-term and daily injections in other studies, which did not result in a decrease in germinal epithelial thickness. It seems that sperm maturation delay due to testosterone level production has been able to lead to a slight increase in germinal epithelium height. The TDI level in the CP, Zn and Vit C groups reached 95%, which is higher compared to the control group (p>0.05).

## Conclusion

Our findings suggest that zinc, which is a cheap supplement, might have possible therapeutic effects in CP-exposed men, and the administration of these micronutrients could be a safe way to improve semen quality and fertility.

## Conflict of Interest

The authors declare that there is no conflict of interest.
